# Development of Systems for the Production of Plant-Derived Biopharmaceuticals

**DOI:** 10.3390/plants9010030

**Published:** 2019-12-24

**Authors:** Ki-Beom Moon, Ji-Sun Park, Youn-Il Park, In-Ja Song, Hyo-Jun Lee, Hye Sun Cho, Jae-Heung Jeon, Hyun-Soon Kim

**Affiliations:** 1 Plant Systems Engineering Research Center, Korea Research Institute of Bioscience and Biotechnology, 125 Gwahak-ro, Yuseong-gu, Daejeon 34141, Korea; irony83@kribb.re.kr (K.-B.M.); pjs12315@kribb.re.kr (J.-S.P.); hyojunlee@kribb.re.kr (H.-J.L.); hscho@kribb.re.kr (H.S.C.); jeonjh@kribb.re.kr (J.-H.J.); 2 Department of Biological Sciences, Chungnam National University, 99 Deahank-ro, Yuseong-gu, Daejeon 34134, Korea; 3 National Research Safety Headquarters, Korea Research Institute of Bioscience and Biotechnology, 30 Yeongudanji-ro, Ochang, Chungbuk-do 28116, Korea; injasong@kribb.re.kr

**Keywords:** molecular farming, plant-derived protein, recombinant protein, expression system, production system

## Abstract

Over the last several decades, plants have been developed as a platform for the production of useful recombinant proteins due to a number of advantages, including rapid production and scalability, the ability to produce unique glycoforms, and the intrinsic safety of food crops. The expression methods used to produce target proteins are divided into stable and transient systems depending on applications that use whole plants or minimally processed forms. In the early stages of research, stable expression systems were mostly used; however, in recent years, transient expression systems have been preferred. The production of the plant itself, which produces recombinant proteins, is currently divided into two major approaches, open-field cultivation and closed-indoor systems. The latter encompasses such regimes as greenhouses, vertical farming units, cell bioreactors, and hydroponic systems. Various aspects of each system will be discussed in this review, which focuses mainly on practical examples and commercially feasible approaches.

## 1. Introduction

The first reports of the production of mammalian proteins in plants appeared in the late 1980s, and from then on the concept of “plant molecular farming” has referred to harnessing the potential of plants as biological factories. The concept of molecular farming or “biopharming” was introduced by Fisher et al. [1,2] to describe “the production of recombinant proteins in plants.” During the early period, the targets of interest (TOI) in this field were mainly recombinant macromolecules, such as blood proteins, vaccines, and antibodies; in addition, cosmetic raw materials and medical therapeutics are now within reach. This review focuses mainly on useful biopharmaceuticals, such as vaccine antigens, antibodies, and industrial enzymes.

Traditional platforms for large-scale production of biopharmaceuticals, based on the bacterium *Escherichia coli*, yeast species, and insect or mammalian cell systems, have been well established and improved over time. The choice of expression/production platform depends not only on an investigator’s need, but also on the final purpose and function of the target biopharmaceuticals. Complex therapeutic proteins, which must be properly folded or processed to achieve the desired degree of biological activity, are produced in yeast or mammalian cell systems rather than in prokaryotes. The biggest concerns in mammalian cell cultures are the high operating costs of scaling up and potential contamination by animal-borne viruses or virions. In light of such concerns, a plant-based production system is a good alternative for producing specific recombinant proteins. Recently, this issue was highlighted when Chinese hamster ovary cells in the production facility for Cerezyme^®^—which is used for treatment of Gaucher disease—were infected with calicivirus. The US Food and Drug Administration (USFDA) quickly authorized Protalix in Israel to undertake commercial production of Elelyso, which was developed in carrot cell culture. More importantly, Elelyso is a good candidate for “biobetter” because, unlike Cerezyme^®^, it harbors terminal mannose residues capable of binding to microphage receptor and so does not require enzyme digestion to cleave inappropriately glycosylated proteins following protein harvest.

**Figure 1:**
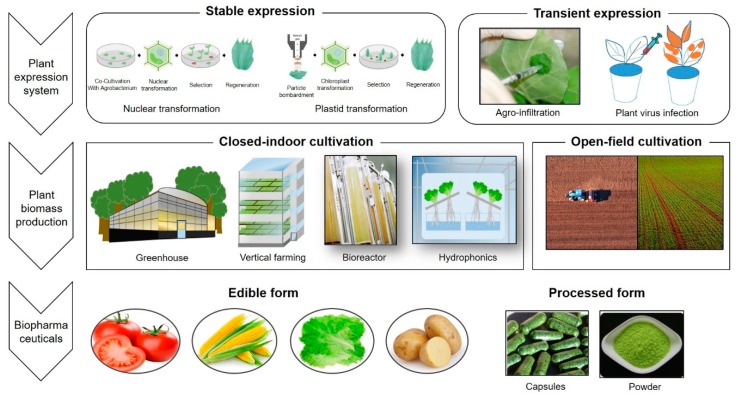
Overview of production system of plant-based biopharmaceuticals and plant biomass.

In here, we review recent advances in plant-based biopharmaceuticals expression platforms and strategies, and on plant biomass production in different facilities (Figure 1). The expression methods used to produce target proteins are categorized as “stable” and “transient” and depend on applications that use whole or minimally processed plants or plant parts. Each type of system can be further subdivided: techniques involving *Agrobacterium*-mediated transformation or particle-bombardment-enabled stable transformation of the nucleus or chloroplast, whereas transient expression may be achieved by using a plant virus or by infiltration by *Agrobacterium* [3,4]. Currently, production of plant biomass expressing the TOI involves two major approaches: open-field cultivation and closed-indoor systems. The open-field simply refers to an outdoor plantation. Because it uses existing agricultural facilities, it is easy to scale-up and its economic benefits can be highlighted. The latter encompasses regimes as greenhouses, plant buildings including vertical farming units, cell bioreactors, and hydroponic systems. Many aspects of each system will be discussed in this review, which focuses mainly on practical examples and commercially feasible approaches.

## 2. Production Systems of Target Proteins of Interests

### 2.1. A “Stable” Expression Platform Using Agrobacterium-Mediated Transformation

The advantages of a “stable” transgenic plant system include high scale-up capacity, unique glycosylation patterns, low risk from animal-borne contaminants, and inexpensive storage costs [5], and it also avoids the need for refrigerated transportation over long distances through local cultivation. To produce valuable recombinant proteins by stable expression system, a lot of plant species, such as tobacco, rice, potato, tomato, and lettuce, have been reported to date (Table 1). Such plants may be grouped into leaf-based and seed-based species. Leafy plants such as lettuce, are used as production platforms with moderately high expression levels: yields of 0.24% [6] and 0.13% [7] have been reported. Leafy non-food crops, such as varieties of tobacco with low nicotine and low alkaloid levels or alfalfa, are particularly suitable hosts because they are perennial plants with high biomass production [8]. Recombinant proteins may be expressed specifically in storage organs or seeds of species such as rice, corn, and potato. Seed or storage-based production platforms are economically viable because they provide almost unlimited capacity, and, due to seed dormancy and storage properties, production of recombinant protein by extraction and purification may be decoupled from crop production [9]. In particular, in terms of downstreaming process, the advantages of seeds having fewer phenolic compounds that may disrupt or strongly deteriorate column resin should be underlined. Previous studies used rice subjected to microprojectile-mediated transformation to produce synthetic human lactoferrin and lysozyme; these proteins made up 0.5% flour weight (25% of total soluble protein (TSP)) and 0.6% of brown rice weight (45% TSP), respectively [10,11]. Unless seed and storage organs are targeted, plants with high water contents (such as tomato and lettuce) are more suitable for molecular farming than dry-tissue plants due to the ease with which proteins are extracted from their tissues [12,13,14]. For instance, thymosin-a1 is easily extracted from tomato fruits with yields of up to 6 μg/g fresh weight [15].

Stable transgenic plants that produce recombinant protein can be generated via two methods: nuclear transformation and plastid transformation (Table 2). Most of the studies mentioned above used stable nuclear transformation; however, expressing recombinant antigens in plant chloroplasts has several advantages, including high levels of expression (up to 72% TSP) [16], extremely high stability [17], and the possibility of open cultivation due to maternal inheritance in most crops [18]. Plastid-based vaccines against many infectious diseases have been developed using plant lines expressing *Mycobacterium tuberculosis* vaccine antigens [19,20,21], *Vibrio cholerae* non-toxin B subunit [22], *Bacillus anthracis* protective antigen [23], *Yersinia pestis* F1-V antigen [24], and *Poliomyelitis* (polio VP1) vaccine antigen [25]. Expression levels ranged from 4% to 18% TSP [26]. It is considered a limitation that chloroplast transformation has been reported only in some plant species, including lettuce [27]. In addition, the fact that the technology has not reached the commercial field means more research is required.

An enormous advantage of using edible plants in such ways is that their tissues can be consumed safely; moreover, oral delivery may induce mucosal immune responses. Although most recent commercial recombinant proteins are produced using transient expression systems, the original concept of producing oral vaccines from transgenic plants containing protein antigens remains viable. Development of representative oral vaccines can be illustrated by the following examples. In 2005, Tanavala et al. [28] conducted a human clinical trial to evaluate the immunogenicity of potato-derived HBsAg and concluded that orally-delivered plant-derived vaccines would help improve global immunization rates. Diseases prevented by mucosal immunity are an important candidate for treatment using oral vaccines. From this perspective, the prefix Muco in the name MucoRice seems to be of great significance. MucoRice-CTB and MucoRice-ARP1 comprise an antigen derived from cholera toxin B (CTB) and the variable domain of a rotavirus-specific lambda heavy-chain antibody fragment, respectively, which accumulate in the storage organelles of rice seeds. Both of these vaccines protect against disease by inducing both mucosal and systemic immunity [29,30].

Potential weaknesses of orally-delivered plant-based recombinant proteins include difficulties in controlling dosage and variable dosage consistency, which may differ from fruit to fruit, plant to plant, and generation to generation. Here are two cases for solving these problems. Protalix Co. uses lyophilized carrot cells containing either anti-TNF or glucocerebrosidase for oral delivery, whereas Interberry, which expresses canine interferon-α, is administered in the form of dried strawberry fruit within dog feed [60].

Despite the merits of transgenic plant-based expression systems, there are still limitations. Mainly, these are related to economic aspects rather than to technical aspects and they are important factors that determine investment by companies working in this field. For example, economic production of plant biomass containing the TOI is the key to commercialization. To overcome these limitations, most production platforms for plant-based recombinant proteins are moving to transient expression systems.

### 2.2. Cell Cultures in “Stable” Systems

Since the first report of albumin production in tobacco cell suspension culture [31], human therapeutic proteins, antigens, scFv, antibodies, and reporter proteins have been introduced to and produced in various plant-derived cell suspension cultures [61]. Use of callus cells to produce a chimeric gene encoding human growth hormone was first reported in sunflower callus tissue [32]; many different types of callus culture have since been developed [62]. Such systems have several advantages, including rapid growth, protein consistency with the use of controlled bioreactors under a contained environment, fewer issues with pathogen contamination involving viral or bacterial toxins, and the ability to address regulatory and environmental concerns regarding the potential release of genetically modified organisms (GMOs) linked to whole-plant systems. In addition, extracting and purifying protein from cell cultures is simpler, more convenient, and more cost-effective than from whole plants, especially when the product is secreted into the culture medium.

Species such as *Nicotiana tabacum*, *N. benthamiana*, *Oryza sativa,* and *Daucus carota* are used most frequently for development of suspension cultures. Recombinant alpha-1-antitrypsin (rAAT) with a secretion signal sequence and sugar starvation-inducible promoter showed the highest rate of production (100–247 mg/L) of transgenic proteins expressed in rice cell suspension culture [33]. Chen et al. reported that bevacizumab, a humanized monoclonal antibody (mAb) targeting vascular endothelial growth factor, produced via a rice callus cell system had similar biological activity, and therefore, might be used in the future as a cost-effective biosimilar treatment [34]. Inducing secretion of target recombinant proteins into the culture medium is a good method for increasing yield or enabling easy harvest and purification. Furthermore, secretion of target protein by cells into the medium is the best choice when production is scaled-up in bioreactors. The signal peptide of α-amylase was used to ensure secretion of recombinant protein by cells [35]. The ER signal peptide facilitated expression and secretion of intracellular rhEPO from hairy root cultures of tobacco (*N. tabacum*), resulting in a yield of up to 66.75 ng/g TSP [36]. In many cases [37,38,39], the promoter and signal peptide from the rice α-amylase gene have been used to develop a two-step process (increasing cell number and maintaining cell viability/activity in the first step and then producing recombinant protein in the second step), resulting in high production of secreted proteins. Although this system has some strengths, it increases both the cost of the process (as it is more labor-intensive) and the risk of contamination when changing the medium. Liu et al. scaled-up production by using a more convenient method based on an air-lift bioreactor, which requires no change of medium [40]. They demonstrated a 6-fold increase in the yield of recombinant HAS using the two-step process.

The approval by the USFDA of a recombinant vaccine against Newcastle disease virus produced in non-nicotinic transgenic tobacco cell cultures was a monumental event in the development of plant cell culture as a bioproduction platform [63]. Since then, the commercial success of Elelyso, the first recombinant pharmaceutical protein for human use produced in plant cells, has proven the value of this approach [64]. *D. carota*, the original species used by Protalix Biotherapeutics, is now the most famous plant species for production of pharmaceuticals, with ten vaccines, against measles, hepatitis B virus (HBV), human immunodeficiency virus, *Y. pestis*, *Chlamydia trachomatis*, *M. tuberculosis*, enterotoxigenic *E. coli*, *Corynebacterium diphtheria*/*Clostridium tetani*/*Bordetella pertussis*, and *Helicobacter pylori*, awaiting completion of development [65].

Rice is one of the most popular plants for the establishment of cell lines in suspension culture. Rice cell suspension cultures may overcome the problem of low expression levels by using the rice α-amylase 3D promoter system. The α-amylase 3D promoter, which is activated by sugar starvation [66], is one of the most widely used metabolite-regulated promoters; indeed, it is used in rice cell suspension cultures to produce various recombinant proteins such as hGM-CSF [41], human growth hormone [42], human VEGF165 [43], FimA mAb [44], bovine trypsin [38], and human pepsinogen C [45].

Plant cell cultures combine the safety of plants with the benefits of a controlled fermenter-based process in a contained cultivation system. A significant issue for plant-based biopharmaceutical manufacturing platforms is the requirement for compatibility with good manufacturing practice (GMP); this is because it is difficult to constrain the entire production chain to a clean-room when using whole plants. The original GMP criteria applied to clinical products produced in bacteria or mammalian cells can be applied directly to plant cells in a bioreactor, but it should be noted that the regulations for biopharmaceuticals produced in whole plants must be changed considerably [67].

To increase the purification efficiency of recombinant proteins produced in plant callus suspension cultures, various tags have been added to appropriate sites within the expression vectors. These include elastin-like polypeptides [46,47], zein-derived peptides [48,49], and hydrophobins [50]. The main characteristic of these tags is that they stabilize the fusion partner and enable accumulation of the fusion protein in discrete storage structures, thereby increasing the yield of the recombinant protein. Expression of green fluorescent protein–hydrophobin fusion (GFP–HFB) proteins increased 2-fold (in comparison with free GFP) upon both transient expressions in *N. benthamiana* leaves [51] and stable expression in tobacco plants [52]. Moreover, a large-scale tobacco BY-2 suspension cell culture increased the yield of a GFP–HFB fusion protein by 3-fold, with good purity and up to 60% recovery [50].

Despite the economic and technical benefits of molecular farming, public and regulatory concerns about containment of GMOs have hindered its widespread adoption, especially in Europe. The European Union has implemented the strictest legislation governing the use of GMOs and GMO-derived products [68] due to concerns about their long-term environmental burden and biosafety. Therefore, the recent trend for the fourth industrial revolution is for transient expression technology to be used in agriculture as a substitute for the creation of new varieties of GMOs [69].

### 2.3. Transient Expression Systems

In the late 1990s, transient expression systems in intact or virally infected plants were considered primarily as a means of checking whether the vector or target protein was expressed, and a means of determining the function of the recombinant protein. Expression would then be moved into a stable transgenic plant system and scaled-up [2]. When the development of edible vaccines that used either the whole or part of a plant proved problematic, various alternative systems were suggested. Transient gene expression is an efficient, time-saving, and widely accepted strategy for producing large amounts of recombinant protein. The advantages of these systems have been well known for the last few decades, and transient gene expression can be detected within a very short time (between 3 h and 6 days). For this reason, many researchers and commercial applications have found transient expression to be a powerful tool. Recent studies describe methodologies that produce milligrams to grams of recombinant protein using a very strong viral replicon vector system [53,54].

Plant culture for transient expression is usually divided into two stages, the pre-inoculation and post-inoculation processes, which require different optimal environments. Cultivation conditions, such as the nutrient composition of the culture medium before agro-infiltration [55] and dehydration of plant material after agro-infiltration [70], are key factors. Changing environmental conditions at appropriate times during each stage may be required to obtain a high yield [56]; a high-nitrate nutrient solution during the pre-inoculation state results in high recombinant protein content [55]. Very recently, interest in the surrounding environment has extended beyond research into the plant itself [57,58,71]. Nitrate-enriched fertilizers that enable plant growth [55], immediate desiccation after agro-infiltration [56], and slightly lower plant density [57], are critical factors for improving plant biomass. Plant density, the number of plants per unit growth area, and the effect of leaf position affect harvested biomass, and are therefore, important factors for achieving economically feasible production [57]. Removal of residual water from a bacterial suspension on detached leaves significantly affects the yield of recombinant hemagglutinin (HA) protein, and recovery from detached leaves in a transient over-expression system is comparable with that from intact leaves [56].

Merlin et al. [59] compared the yields of optimized target proteins after downstream processing in three different plant expression systems and reported that the highest yields of an optimized variant protein were achieved in a transient expression system using the MagnICON vector. They hypothesized that a significant increase in the yield of the TOI could be achieved using transient expression.

## 3. Plant Biomass Production Systems

Since the 1990s, many foreign genes related to vaccine antigens, antibodies, and therapeutic proteins have been introduced into plant genomes, resulting in high-value transgenic plants. Different types of production systems for various plant-based recombinant proteins have been reviewed above. This next section will review and discuss two different approaches to cultivating plant itself expressing various recombinant proteins: open-field cultivation and closed culture systems.

### 3.1. Open-Field Cultivation

Molecular pharming of entire plants involves genetic engineering to insert genes encoding useful pharmaceuticals. The open-field approach does not require expensive infrastructure for plant production and allows purification capacity to be quickly scaled to meet demand, meaning costs of the whole process are greatly reduced. When cultivating any given species in the field, however, the potential risk of cross-pollination must be considered. Rice, wheat, and peas are all self-pollinating plants, but the residual risk of out-crossing to wild relatives still needs to be assessed [72]. Tobacco plants are favored for the production of biopharmaceuticals due to their fast growth, rapid reproduction, maintenance of genetic stability, and non-feed crops [73,74]. Although tobacco species are cross-pollinating, harvesting the plants prior to flowering can prevent or minimize the risk of contamination.

Devos et al. [75] demonstrated that to minimize vertical gene flow from GM oilseed rape to wild-type plants in the field, critical practical measures, including the use of certified seed, separation of fields, harvesting the crop at the correct developmental stage, control of self-seeding in subsequent crops, and accurate record-keeping, are required. These proposed items are supported by the important cases described below.

Chicken egg-white avidin was developed by ProdiGene and marketed by Sigma-Aldrich (product number A8706) for use as a diagnostic reagent. Despite the use of a stable expression system, transgenic maize seeds produced very high yields of over 2% [76]. Reports suggest that compared with conventional extraction from eggs, this method provided a 10-fold saving in the cost of starting materials. The transgenic maize was planted in a greenhouse or in open fields for trait analysis and maintained by out-crossing and segregation of the T1 through T4 generation. However, when the transgenic crop was grown and harvested in a field trial in Nebraska, some of the GM kernels remained on the ground and germinated the following year in the same field in which soybeans were subsequently grown. In another case involving the same company, GM pharma corn grown in Iowa was cross-pollinated by crops grown nearby [77], resulting in wholesale destruction of potentially contaminated plants.

These incidents had a negative effect on future research. Afterwards, an increase in public opposition to GM crops producing pharmaceutical components led to stronger regulation and withdrawal of investment by big biotech companies such as Monsanto. Due to the very strict guidelines governing cultivation of plants expressing foreign genes of interest, which are referred to as living modified organisms, there are no examples of “approved” cultivation of transgenic plants producing pharmaceutical proteins anywhere in the world; this is because the number of permits for field trials of such crops issued by the USFDA dropped sharply after 2000.

A positive case of a seed-based platform in open-field cultivation is one developed by SemBioSys Genetics Inc. Safflower seed-based recombinant insulin produced at levels >1% of total seed protein, was tested successfully in human clinical trials, and bioequivalence to commercial insulin was demonstrated [78]. Open-field growth is one of the most realistic options for large-scale production of transgenic seed crops. In 2008, cultivation of safflower was scaled-up to 1 tonne of seed per acre and then subjected to a pilot scale process; advanced technology now allows production of up to 2–4 kg of recombinant protein per hectare, depending on the molecular mass of the target protein.

When plant-made pharmaceuticals produced via molecular farming enter the commercial market, their production needs to meet regulatory requirements and address public concerns regarding containment of transgenic crops [79]. Best management practices to reduce seed-mediated gene flow and environment contamination by pollen transmission have been published [80]. Two recent articles indicate that the incidence of pollen transmission ranges from 0.00024% to 0.0087% [81,82].

After approval by the USFDA Animal and Plant Health Inspection Service, tobacco plants producing biologically effective interferon-α2b (IFN-α2b) for treatment of hepatitis C infection were grown over an area of approximately 0.26 acres [83]. Expression levels reached 20% of TSP, but only about 87.2 g of IFN-α2b was generated after a single harvest of tobacco plants grown in the field; this was because harvesting biomass is performed by taking the young leaves of premature flowering plants. Thus, a chloroplast-based production system is considered to be a suitable method for cultivation in the field, due to advantages such as minimizing transmission of transgenes via pollen spread.

Ventria Bioscience (www.ventria.com) used field-grown rice to produce human lactoferrin [11] and lysozyme [10] for treatment of acute diarrhea and dehydration. A randomized, double-blind, controlled trial of a rice-based oral rehydration solution containing these two biopharmaceuticals was conducted in children and the results demonstrated beneficial effects [84]. Also, it was reported recently that VEN120, the trade name for recombinant human lactoferrin derived from rice, reduces inflammation, and promotes immune self-tolerance [85].

### 3.2. Closed Culture Systems

Due to issues with field cultivation of GMO, most transgenic plants grown for molecular pharming are cultivated in “closed systems” (a simplified term for “closed plant production system”). The various categories of closed culture system are discussed in detail below. Such systems have been widely used in Japan for commercial purposes since 2002 [86]. Kozai pointed out several advantages of closed systems: first, rapid and efficient growth due to optimized growth conditions; second, the significantly higher quality of plants due to uniform pest control, an environment free of insects, pathogens, and weather disturbances; third, higher productivity achieved by using multi-layered shelves, a high planting density, and a shorter production period; and, lastly, easier control of plant development.

Farran et al. [87] harvested 4.2 mg of recombinant histidine-tagged human cardiotrophin (rhCT-1) from a single plantlet grown for 20 days in a walk-in room; they calculated yields of up to 2.5 kg rhCT-1 per unit of a commercial-type closed system per year. Furthermore, it was suggested that yields of 3.2 kg rhCT-1 would be possible by increasing the harvesting frequency once per week. This would generate an amount of protein that far exceeds the amount required worldwide.

In conclusion, a confined or closed system involving transient agro-infiltration is an environmentally friendly method of producing recombinant proteins, which even in seeding plants avoids the potential hazards of pollen or seed spread.

#### 3.2.1. Greenhouse Systems

A greenhouse is an example of an “open plant production system” (simplified to “open system”). It is less well controlled than a closed system because exchange of heat energy, CO_2_, and water may occur between its interior and the external environment [86]. The advantage of a greenhouse plant production system over open-field production is a higher biomass yield due to technological improvements that have, over the past two decades, resulted in a 15-fold increase in productivity [88,89]. Greenhouses can be designed to function as containment facilities that prevent escape of transgenes [74]; they also maximize protein productivity by optimizing conditions for plant growth, development, and accumulation of nutritional compounds. Controlled environments provide an opportunity to increase production of “value-added” crops containing high concentrations of phytochemicals, such as lycopene in tomato [90]. In addition, stable transgenic plants produced by *Agrobacterium*-mediated transformation are usually grown in containment systems such as greenhouses. An efficient biomass production system for transgenic lettuce harboring HBV surface antigens for use as anti-HBV oral vaccines [91] and transgenic tobacco producing an anti-HBsAg plantibody [92] was developed in a year-round cropping greenhouse to optimize product processing; this was a step towards manufacture of a standardized oral vaccine with reliable efficacy. In general, these transgenic plants were seeded initially in indoor floating beds and then transferred to greenhouses for further growth. In other cases, seeds of transgenic Petit Havana tobacco plants expressing IFN-α2b were propagated in a greenhouse suitable for field transplantation [83].

Fraunhofer IME, a leading German company working in this field, grows transgenic tobacco plants expressing P2G12 [93] (used in phase I progress trials) according to GMP guidelines in a specialized containment greenhouse in Aachen. ORF Genetics (http://www.orfgenetics.com/) produces endotoxin-free growth factors and cytokines in barley grains grown in their own geothermal high-tech greenhouse [94]. Up to 130,000 bio-engineered barley plants at a time may be grown for 90 days; these plants are a remarkable source of commercialized products, such as Bioeffect^TM^ EGF and ISOKINE^TM^.

Greenhouses are one of the most promising systems for large-scale production of biomaterials in plants as they provide a compromise between capital investment and ease of scale-up.

#### 3.2.2. Bioreactor Culture Systems

Suspension culture systems have a long history, having been used since 1902 [95]. They are employed widely in the plant biotechnology field as a convenient tool for large-scale production of recombinant proteins, secondary metabolites, and other pharmaceutical ingredients [96]. Conditions are controlled easily, which enables more consistent yields; moreover, secretion of target materials into the medium makes it possible to simplify the procedure and reduce costs, thereby providing significant advantages over methods based on extracting and purifying target material from plant cells. Due to these advantages, many cases of plant suspension culture have been reported; proteins produced using this method include the HN protein of Newcastle disease virus, human glucocerebrosidase, recombinant α-galactosidase-A, and anti-tumor necrosis factor antibodies [96]. To optimize important factors that affect suspension culture, one must consider selection of the host plant, the type of plant material (e.g., callus or hairy root), media components, and the bioreactor type and operating method. These considerations are similar to those for well-characterized production systems based on microbial or mammalian cells [97].

Bioreactor operations are critical for the successful development of large-scale production processes [61]. There are several types of bioreactor, including air-lift reactors, bubble column reactors, membrane reactors, rotating drum reactors, single-use bubble column reactors, stirred tank reactors, wave reactors, wave and undertow reactors, and bench-top bioreactors [98]. Overall, their volumetric productivity ranges from 4.5–7.7 mg/L [99] to 100–247 mg/L [33]. Temporary immersion bioreactors, which immerse biomass in liquid media periodically, are amenable to scaled-up automated micropropagation of large quantities of shoot biomass under standardized conditions [100]. Using this system, fragment C of tetanus toxin accumulated to about 95 mg/L (8% TSP) [101], and outer surface protein A of *Borrelia burgdorferi* (OspA) gave a yield of 108 mg/L (7.6% TSP) [102]. An air-lift type bioreactor (130 L) is another efficient system used to produce recombinant proteins such as the B subunit of *E. coli* heat-labile toxin, which accumulated to 0.36% TSP in Siberian ginseng somatic embryos [103]. When expression of tomato bifunctional nuclease1 was compared between *N. benthamiana* and BY-2 cell suspension cultures, the suspension culture showed stable maintenance with lower expression than in *N. benthamiana* [104]. The most impressive achievement to date from a cell culture system using a tank bioreactor is the first plant-produced commercial therapeutic protein, produced in carrot cells grown in a flexible polyethylene disposable bioreactor (www.protalix.com).

This system is easy to use, requires a low initial capital investment, and can be scaled-up cost-effectively. NBM, a Korean venture company operating in the field of molecular farming, has adopted the flexible polyethylene disposable bioreactor to express recombinant protein in rice cells due to the easy control of culture conditions, rapid growth, and upscaling potential. Recombinant proteins are secreted into the culture medium, thereby simplifying isolation and purification of the products (www.nbms.co.kr). NBM has launched serum-free, animal component-free, and endotoxin-free INNOkine and INNOzyme products for generation of stem cell-related growth factors, bioreagents, cosmetic ingredients, and cosmetics.

However, this system also has disadvantages. A typical problem is management of contamination when subculturing. Using bioreactors for plant cell or hairy root culture requires cultivation techniques similar to those used for mammalian cell culture systems, which involve sterilization; therefore, it is difficult to expand such systems to very-large-scale production of 250,000 L or more [105]. Although the set-up phase is shorter for suspension cultures than for stable transgenic plants, the scale-up and maintenance of very-large-scale production require specialized equipment and intensive labor [72]. Estimates of costs of the two systems suggest that 1 kg of grain costs about US $0.20 to produce, but the total production costs of 1 kg of plant biomass in a laboratory-scale reactor are about US $200 when labor, special equipment, and consumables are considered.

Recently, the biomanufacturing industry has widely accepted disposable bioreactor systems such as wave-mixed bioreactors or stirred tank reactors for plant cell cultures as an alternative to the stainless steel bioreactors used for mammalian cell cultures due to their cost-effectiveness, flexibility, and safety [106,107,108]. Tobacco BY-2 cells are a representative platform for the manufacture of biopharmaceutical proteins in suspension culture. Raven et al. [109] succeeded in cultivating BY-2 suspension cells secreting a human M12 antibody in a disposable orbital shaker at a working volume of 100 L; this scale is 200-fold greater than that used for routine cultivation in shake flasks. A manufacturing process involving expanded bed adsorption chromatography showed high yield recovery (ranging from 75% to 85%) and product purity >95%. A little earlier, a study to determine the suitability of the orbital shaker disposable SB200-X bioreactor system for scaled-up cultivation of BY-2 cells secreting human IgG reported cell growth and recombinant protein produced yields comparable with those obtained after cultivation in 500 mL shake flasks [110]. Semi-continuous operation through two phases each of growth and expression was used for production of an active tetrameric form of recombinant butyrylcholinesterase (BChE), a large and complex human enzyme, from a transgenic rice cell suspension bioreactor; the maximum yield was 1.6 mg BChE/L [111].

Despite advantages such as cost-effectiveness, rapid scale-up, and low risk of pyrogen contamination, only one therapeutic product has entered the market: glucocerebrosidase, which is produced in Israel by Protalix.

#### 3.2.3. Hydroponic Systems

An indoor hydroponic system guarantees fast growth, is free from soil-borne disease, and does not require pesticides or herbicides. Hydroponic systems can be adopted to grow plants stably or transiently expressing transgenes in a greenhouse or a closed growth system. A fully contained hydroponic system used for primary research was used to grow vacuum-infiltrated *N. benthamiana* plants transiently expressing recombinant proteins [112]. Several proprietary improvements have allowed this plant platform to be adapted to generate a large-scale automated hydroponic growth system.

Both tomato plants expressing the F1-V antigens of bubonic plague and the greenhouse-adapted wild-type plants were grown hydroponically for 24–30 weeks in a greenhouse equipped with heating and evaporative cooling systems [113]. Plant growth, cumulative fruit yield, fruit TSP concentration, and cumulative TSP production were measured. Cumulative fruit yield per plant of F1-V tomato over 13 weeks of harvest was almost half that of the greenhouse-adapted wild-type; however, the TSP concentration in the fruit was at least three times higher. Therefore, the critical metric to consider when using hydroponic cultivation for high-value protein production is not biomass productivity but the protein concentration in fruit. The following studies were conducted to determine optimal conditions for hydroponic cultivation of biopharmaceuticals. Transgenic tomato plants expressing F1-V protein were grown hydroponically at high electrical conductivity (EC) in nutrient solution; increasing EC led to a significant fall in the levels of both TSP and F1-V antigen in the fruit [114]. To identify the optimum time of harvest to maximize the yield of F1-V antigen, protein levels in the green fruit were investigated at different developmental stages [115]. Harvesting small green fruits without pruning was the most practical way to maximize antigen yield.

In another study, transgenic lettuce plants expressing coagulation factor IX fused to a CTB carrier were grown in a scaled-up Fraunhofer cGMP hydroponic system and harvested at about 870 kg fresh weight per 1000 ft^2^ per annum, yielding 24,000–36,000 doses [116]. This hydroponic system consisted of two wire shelving units with four growing areas per rack. Growth conditions were as follows: light intensity, 70–90 mmol m^−2^ s^−1^; photoperiod, 18 h; temperature, 23–26 °C, and humidity, 20–60%. This scalable production method that is translatable to cGMP is performed well using a transformed edible crop.

To ensure GMP, a rice-based oral vaccine against cholera, MucoRice-CTB, was produced in a closed hydroponic cultivation system, thereby yielding a product that met regulatory requirements [117]. These plants were cultivated using hydroponic techniques based on a circulating nutrient solution and a polystyrene foam board as a floating holder in a molecular farming factory that can be harvested three times a year. To ensure batch-to-batch consistency with respect to plant growth and CTB expression, appropriate environmental conditions for growing the rice, including lighting, wind velocity, temperature, humidity, and nutrient solution, were established.

Hydroponic systems are attractive due to their high recycling efficiency and ease with which fertilization and root temperature can be controlled. Consequently, these systems can be a good choice for cultivation of pharmacological plants (not only leafy vegetables but also fruit or storage vegetables). In Japan, the Hokkaido Center of the National Institute of Advanced Industrial Science and Technology invested in commercialization of a medicine sourced from strawberries expressing canine interferon, which suppresses gum inflammation in dogs. The strawberries were grown in a previously established closed-type plant factory equipped with a hydroponic cultivation bed [118]. Such a fully closed hydroponics system can be operated year-round, yielding several harvests annually; thus, it is both cost and production-effective.

## 4. Conclusions

There are various systems for producing industrially or pharmaceutically useful recombinant protein in plants, and each system has strengths and weaknesses. We have reviewed many examples of production using each system. In the future, demands for plant-based biopharmaceuticals will increase. Most importantly, the economic feasibility of producing useful recombinant proteins will determine the viability of companies that have just started industrializing this process. Any successful strategy must make good use of the advantages of producing “bio-betters” by selecting suitable methodologies to overcome the low price of mass production in *E. coli* and the excellent efficacy of animal cells. Of the production systems discussed in this review, transient expression systems have been most frequently adopted by different companies, with 16 market-releasable items that are safe and environmentally friendly currently in indoor or outdoor trials [119]. Bioreactor-based cell culture systems, which can be rapidly scaled-up and are free of mammalian pathogens, have been adopted by traditional fermentation-based companies. Despite this, the situation concerning GM plants is still not always favorable and some major hurdles remain. The final goal, namely, the production of a plant-based vaccine in fresh plant materials that can be eaten, is yet to be achieved. Given market prices, the large capacity required, and the general understanding of plant biopharmaceuticals by the general population, it is difficult to challenge the dominance of conventional production platforms based on *E. coli*, mammalian cells, and yeast. However, recent advances in plant molecular farming allow very large-scale, even multi-tonne scale, production of plant-based pharmaceuticals. The positive trend is that many researchers try to start a venture based on technology acquired from the laboratory. Thus, technologies from various fields will converge and continue to develop; eventually, clinical products produced using these systems will enter the market.

## Figures and Tables

**Table 1:** Published studies on production systems of target proteins.

Expression System	Host Plant	Protein Yield	Target	Material	Binary Vector/Agrobacterium Strain	Ref.
*Agrobacterium*-mediated transformation	Lettuce	0.24%(*w*/*w*, TSP)	cholera toxin B subunit (CTB)	Cotyledons	pMYO114/LBA4404	[6]
	Lettuce	0.13%(*w*/*w*, TSP)	pro-insulin (Pins)	Cotyledons	pCAMINS/LBA4404	[7]
	Tomato	6 ug/g fresh weight	thymosin α1	Cotyledons/hypocotyls	PG-pRD12-4×Tα1/EHA105	[15]
	Tobacco	4% of TSP	TBAg-ELP	Leaf disk	pCB301/C58C1	[19]
	Carrot	0.056% of TSP	ESAT6	Zygotic embryos	pBI121/-	[21]
	Carrot	0.002% of TSP	CFP10	Zygotic embryos	pBI121/-	[21]
	Potato	8.5 μg/g FW	HBsAg	Leaf disk	pHB114/LBA4404	[28]
	Rice	0.15% of seed weight	CTB	Seed	pGPTV-35S-HPT/LBA4404	[29]
	Rice	11.9% of total protein	ARP1	Seed	pZH2Bik45G1B/-	[30]
Microprojectile-mediated transformation	Rice	0.6% (*w*/*w*, 45% of TSP)	Glutelin1 (Gt-1)	Embryogenic callus	pAPI134/-	[10]
	Rice	0.5% (*w*/*w*, 25% of TSP)	hLF	Embryogenic callus	pCRGT1/-	[11]
	Lettuce	>72% of TSP	CTB-Pins	Plastid	pBSSK+/-	[16]
	Tobacco	>70% of TSP	plyGBS	Plastid	pRB95/-	[17]
	Tobacco	-	EPSPS	Plastid	pZS-RD/-	[18]
	Tobacco	7.5% of TSP	CTB	Plastid	pLsDV/-	[20]
	Lettuce	0.75% of TP	CTB	Plastid	pLsDV/-	[20]
	Tobacco	4.1% of TSP	CTB	Plastid	pLD-CtV2/-	[22]
	Tobacco	14.2% of TSP	Protective antigen	Plastid	pLD-ctv/-	[23]
	Tobacco	3.68% of TSP	F1-V	Plastid	pLDS-F1V/-	[24]
	Tobacco	4–5% of total leaf protein	CTB-VP1	Plastid	pGEM-T/-	[25]
Cell and tissue culture	Tobacco	0.25 ug/mg protein	Human serum albumin (HSA)	Leaf disc	pMOG18/GV2260	[31]
	Sunflower	0.02% of hGH transcripts	human growth hormone (hGH)	Callus	pRK290/A208	[32]
	Rice	10% of TSP	α-1-antitrypsin	Callus	pAPI73/-	[33]
	Rice	242.8 mg/kg FW	mAb	Callus	pUN1390/EHA105	[34]
	Rice	699.79 ng/g FW	Interferon-gamma	Callus	pBS3S/LBA4404	[35]
	Tobacco	185.48 pg/g FW	rhEPO	Hairy root	pK7WG2D/A. rhizogenes	[36]
	Rice	76.5 mg/L	HSA	Callus	pBluescript SKII+/EHA105	[37]
	Rice	15 mg/L	Trypsin	Callus	pMYT111/LBA4404	[38]
	Rice	31.4 mg/L	human cytotoxic T-lymphocyte antigen 4-immunoglobulin	Callus	pMYN409/-	[39]
	Rice	45 mg/L	HSA	Callus	pA3HSA/EHA105	[40]
	Rice	73 mg/g cells	human granulocyte-macrophage colony stimulating factor	Callus	pMYN24/-	[41]
	Rice	57 mg/L	hGH	Callus	pMYN449/-	[42]
	Rice	19 mg/L	rhVEGF165	Callus	pMYD171/-	[43]
	Rice	17.3 mg/L	FimA mAb	Callus	pMYV657/-	[44]
	Rice	18 mg/L	human pepsinogen C	Callus	pMYD213/-	[45]
	Tobacco	11% of TSP	Elastin-like polypeptides (ELPs)	Leaves	pCaMterX/EHA105	[46]
	Tobacco	6.42 mg/kg FW	ELPs	BY-2 cells	pCaMterX/EHA105	[47]
	Tobacco	-	Zein -derived peptides	Leaves	pC2300/EHA105	[48]
	Tobacco	-	Zein -derived peptides	Leaves	pBin19/EHA105	[49]
	Tobacco	0.30 ± 0.018 g/L	Hydrophobins	BY-2 cells	pCaMterX/EHA105	[50]
	Tobacco	5.0 mg/g FW	Hydrophobins-GFP	BY-2 cells	pCaMterX/EHA105	[51]
	Tobacco	0.2% of TSP	ELP/HFBI	Leaves	pCaMterX/-	[52]
Transient expression system	Tobacco	0.5 mg/g FW	mAb	Leaves	pBY/LBA4404	[53]
	Tobacco	1.0 mg/g FW	BMVCP/CMVCP/MRFVCP	Leaves	pBYR2fp/GV3101	[54]
	Tobacco	>1.0 mg/g FW	Hemagglutinin (HA)	Leaves	pNM216/GV3101::pMP90	[55]
	Tobacco	846 ug/g FW	Hemagglutinin (HA)	Leaves	pNM216/GV3101::pMP90	[56]
	Tobacco	215 ug/g fresh mass	Hemagglutinin (HA)	Leaves	pNM216/GV3101::pMP90	[57]
	Tobacco	2.0 ug/mg TSP	rhEPO	Leaves	pEG101/EHA105	[58]
	Tobacco	226.9 µg/g FW	human glutamic acid decarboxylase	Leaves	pK7WG2/EHA105	[59]

**Table 2:** Summarizing the advantages and disadvantages of plant expression systems.

Expression System	Advantages	Disadvantages
Production System of Target Proteins		
Nuclear stable transformation	- High scale-up capacity - Unique glycosylation pattern - Low risk from animal-borne contaminants - Inexpensive storage costs - Applied and reported in many plant species	- Dosage control and dose consistency of orally-delivered plant-based recombinant proteins - Limitations of economic aspects of plant biomass production including TOI
Plastid stable transformation	- High levels of expression - Very high stability - Potential open cultivation by maternal inheritance	- Lack of research for commercial applications - Limitation of vaccines production in only some plant species
Transient expression	- Efficient, time-saving, and widely accepted strategy for producing large amounts of recombinant protein - Induce gene expression in a very short time (3 h–6 days) - High levels of expression: potential production of Gram levels. - Economic efficiency and commercialization of recombinant proteins	- Effect of biomass on plant density, growth area, and leaf position - Yield changes in recombinant protein yields by various factors
Plant Biomass Production Systems		
Open field cultivation	- Low infrastructure cost - Rapid production capacity expansion - The most realistic option for large-scale production	- Potential risk of cross-pollination - Very strict guidelines governing cultivation of transgenic plants - Need to address GMO regulatory requirements and public concerns
Closed culture	- Fast and efficient growth by optimized growth conditions - Cost-effectiveness and rapid scale-up - Significantly improved plant quality in a controlled environment - high productivity with multi-layer shelves, a high planting density, and a short production period - Easy control of plant development - Prevention the potential hazards of pollen or seed spread - Possible to grow transgenic plants expressing transgenes	- Various important factors affecting suspension culture - Possibility of contamination when subculturing in a bioreactor culture system - Limitations of large-scale production (over 250,000 L) expansion in bioreactor culture systems - Low commercial application of therapeutic products
